# A global transcriptional analysis of *Plasmodium falciparum *malaria reveals a novel family of telomere-associated lncRNAs

**DOI:** 10.1186/gb-2011-12-6-r56

**Published:** 2011-06-20

**Authors:** Kate M Broadbent, Daniel Park, Ashley R Wolf, Daria Van Tyne, Jennifer S Sims, Ulf Ribacke, Sarah Volkman, Manoj Duraisingh, Dyann Wirth, Pardis C Sabeti, John L Rinn

**Affiliations:** 1 Department of Systems Biology, Harvard Medical School, 200 Longwood Avenue, Boston, MA 02115, USA; 2 Broad Institute, 7 Cambridge Center, Cambridge, MA 02142, USA; 3 Department of Organismic and Evolutionary Biology, Harvard University, 26 Oxford Street, Cambridge, MA 02138, USA; 4 Department of Immunology and Infectious Diseases, Harvard School of Public Health, 651 Huntington Avenue, Boston, MA 02115, USA; 5 School of Nursing and Health Sciences, Simmons College, 300 The Fenway, Boston, MA 02115, USA; 6 FAS Center for Systems Biology, Harvard University, 52 Oxford Street, Cambridge, MA 02138, USA; 7 Beth Israel Deaconess Medical Center, 330 Brookline Avenue, Boston, MA 02215, USA; 8 Department of Stem Cell and Regenerative Biology, Harvard University, 7 Divinity Avenue, Cambridge, MA 02138, USA

## Abstract

**Background:**

Mounting evidence suggests a major role for epigenetic feedback in *Plasmodium falciparum *transcriptional regulation. Long non-coding RNAs (lncRNAs) have recently emerged as a new paradigm in epigenetic remodeling. We therefore set out to investigate putative roles for lncRNAs in *P. falciparum *transcriptional regulation.

**Results:**

We used a high-resolution DNA tiling microarray to survey transcriptional activity across 22.6% of the *P. falciparum *strain 3D7 genome. We identified 872 protein-coding genes and 60 putative *P. falciparum *lncRNAs under developmental regulation during the parasite's pathogenic human blood stage. Further characterization of lncRNA candidates led to the discovery of an intriguing family of lncRNA telomere-associated repetitive element transcripts, termed lncRNA-TARE. We have quantified lncRNA-TARE expression at 15 distinct chromosome ends and mapped putative transcriptional start and termination sites of lncRNA-TARE loci. Remarkably, we observed coordinated and stage-specific expression of lncRNA-TARE on all chromosome ends tested, and two dominant transcripts of approximately 1.5 kb and 3.1 kb transcribed towards the telomere.

**Conclusions:**

We have characterized a family of 22 telomere-associated lncRNAs in *P. falciparum*. Homologous lncRNA-TARE loci are coordinately expressed after parasite DNA replication, and are poised to play an important role in *P. falciparum *telomere maintenance, virulence gene regulation, and potentially other processes of parasite chromosome end biology. Further study of lncRNA-TARE and other promising lncRNA candidates may provide mechanistic insight into *P. falciparum *transcriptional regulation.

## Background

The causative agent of the most severe form of human malaria, *Plasmodium falciparum*, is a unicellular eukaryotic parasite transmitted through the bites of infected mosquitoes. The most vulnerable population to malarial disease is African children, but a staggering 3.3 billion people - half the world's population - are at risk for malarial infection. Despite recent research advances [1-6], the mechanisms *P. falciparum *utilizes to regulate mutually exclusive expression of multi-gene virulence families and stage-specific expression of approximately 80% of its genome during pathogenic blood stage development remain elusive.

Most confounding is the scarcity of sequence-specific transcription factors and *cis*-acting regulatory elements, coupled with the apparent lack of both RNA interference machinery and DNA methylation in the parasite [7,8]. However, the recent discovery of an expanded lineage of 27 ApiAP2 (apicomplexan apetela 2) transcription factors may partially explain how the parasite regulates its unusual genome [9,10]. Additionally, it is becoming increasingly clear that chromatin remodeling and epigenetic memory play an important role in blood stage-specific expression and antigenic variation of virulence genes [6,11,12]. Notably, while the parasite lacks many of the conventional regulatory mechanisms of other organisms, it has a full arsenal of conserved histone modifying enzymes, and a higher than average number of RNA-binding proteins [8,13].

In eukaryotes spanning from yeast to humans, epigenetic regulation incorporates feedback from non-coding RNAs. Specifically, long non-coding RNAs (lncRNAs) and small non-coding RNAs often interface with RNA binding proteins and chromatin remodeling complexes to modulate their targeted genomic loci [14-18]. For example, in × chromosome inactivation at least seven distinct lncRNAs coordinate the selection and silencing of an entire chromosome [19]. As another example, long telomeric repeat-containing RNA (TERRA) transcripts have been recently discovered as a major constituent of telomeric heterochromatin. TERRA interacts with telomere-associated proteins such as telomerase, is developmentally regulated, and is implicated in telomere replication and structural maintenance processes [20-25].

To investigate putative regulatory roles for lncRNAs in *P. falciparum*, we designed a high-resolution DNA tiling array to survey transcriptional activity during the parasite's pathogenic human blood stage. We identified 60 lncRNA candidates and characterized their G+C content, evolutionary conservation, expression profile, and correlation with neighboring genes. Notably, our transcriptional profiling and subsequent analysis revealed an outlier on all fronts: a long telomere-associated non-coding RNA gene, termed lncRNA-TARE-4L, encoded in the telomere-associated repetitive element (TARE) tract of chromosome four.

Upon further investigation of the lncRNA-TARE-4L locus, we discovered a multi-gene family of lncRNA-TAREs. We have mapped homologous lncRNA-TARE loci on 22 of 28 *P. falciparum *chromosome ends, and quantified the coordinated, stage-specific transcription of 15 distinct lncRNA-TARE sequences using quantitative real-time PCR (qRT-PCR). We additionally employed rapid amplification of cDNA ends (RACE) to map putative transcriptional start and termination sites of lncRNA-TARE genes, including three sequences not investigated by qRT-PCR. Our RACE results suggested two dominant transcripts of approximately 1.5 kb and 3.1 kb are transcribed from the TARE 3 boundary towards the telomere. Interestingly, we also found that an upstream sequence type B (upsB-type) *var *virulence gene is adjacent to each predicted lncRNA-TARE gene and that lncRNA-TARE sequence is enriched with transcription factor binding sites only otherwise found in upsB-type *var *gene promoters.

Our results complement the recent *P. falciparum *transcriptome studies of Otto *et al*. [5], Raabe *et al*. [26], and others by providing stage-specific profiling and characterization of several previously unidentified *P. falciparum *lncRNA candidates, including a long telomere-associated non-coding RNA family. Specifically, we have demonstrated that long telomere-associated lncRNAs are coordinately expressed after parasite DNA replication from at least 18 chromosome ends. Taken together, this work provides new insights into *P. falciparum *non-coding RNA transcription and contributes a previously uncharacterized parallel between *P. falciparum *and model eukaryote chromosome end biology.

## Results

### Tiling microarray experimental design

In order to comprehensively identify and characterize long non-coding transcripts in *P. falciparum*, we selected overlapping probes tiling approximately 22.6% of the *P. falciparum *genome at 12-bp median resolution. Notably, our DNA tiling array design provides over one order of magnitude denser probe coverage than previous *P. falciparum *transcriptional profiling arrays, and is unique in that it deeply samples both genic and intergenic sequence [4]. Probes cover 561 Watson (+) strand protein-coding genes, 699 Crick (-) strand protein-coding genes, two ribosomal RNA genes, and 1.73 Mb of intergenic sequence on *P. falciparum *chromosomes 2, 3*, 4, 5*, 7, 9, 12* (asterisks indicates partial coverage; see Materials and methods and Additional file 1 for further genome coverage details).

We harvested RNA from highly synchronous 3D7 parasites to capture global transcriptional changes during the parasite's two major intraerythrocytic developmental cycle transitions: ring to trophozoite and trophozoite to schizont stage. During the *P. falciparum *intraerythrocytic developmental cycle, the parasite first exports cytoadherence surface proteins to sequester itself in host tissue (ring stage = 0 to 24 hours). This is followed by hemoglobin digestion and DNA replication (trophozoite stage = 24 to 36 hours), and, finally, nuclei segmentation and formation of 16 to 32 daughter merozoites (schizont stage = 36 to 48 hours) [27,28]. Specifically, we profiled the polyadenylated RNA population transcribed from both genomic strands at 18 (ring), 24 (ring/trophozoite), 30 (trophozoite), and 36 (trophozoite/schizont) ± 3 hours post-erythrocyte invasion (hpi) using our custom DNA tiling microarray.

### Identification of lncRNA candidates

To identify transcriptionally active regions (TARs) along the *P. falciparum *genome, we analyzed normalized probe hybridization intensities using a window-based scan statistic algorithm. As proposed by Guttman *et al*. [29,30], a TAR can be reasonably defined as a contiguous stretch of tiling probes with signal intensity significantly above the background distribution of permuted hybridization intensities. Briefly, we calculated a scan statistic score for iterative window intervals, controlling the probability of one or more intervals being erroneously called significant at 5% [31]. As the final step, we merged overlapping intervals to define TAR boundaries. Taken together, this highly conservative approach corrects for multiple testing and provides strong family-wise type I error rate control in our set of significant TARs.

**Figure 1:**
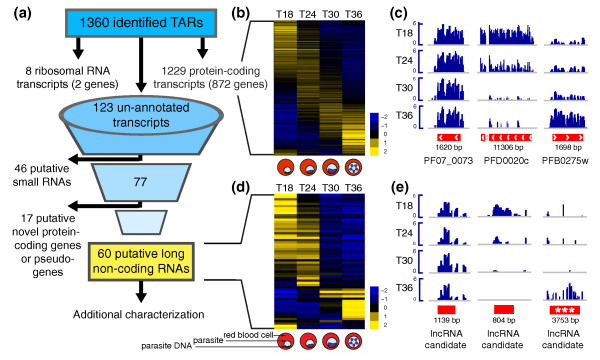
**Global transcriptional profiling reveals 60 putative *P. falciparum *long non-coding RNAs under developmental regulation**. **(a) **We identified 1,360 TARs along the *P. falciparum *genome, of which 1,229, 8, and 123 predicted transcripts exhibit overlap with known protein-coding genes, ribosomal RNA genes, and un-annotated intergenic regions, respectively. Further filtering of un-annotated transcripts for putative lncRNAs eliminated 46 putative small RNAs and 17 putative novel protein-coding genes or pseudogenes, leaving a set of 60 putative lncRNAs for additional characterization. **(b,d) **Protein-coding and putative lncRNA transcripts follow the global expression cascade expected during the *P. falciparum *intraerythrocytic developmental cycle. The mean log2 probe hybridization for each transcript is plotted from time-points T18, T24, T30, and T36 ± 3 hpi corresponding to the ring, ring/trophozoite, trophozoite, and trophozoite/schizont intraerythrocytic stages of parasite growth (pictured below each column). Rows are mean centered. **(c,e) **Housekeeping gene seryl-tRNA synthetase (PF07_0073), early stage marker (PFD0020c), and late stage marker (PFB0275w) exhibit expected differential expression patterns. Examples of putative lncRNAs are also shown, including lncRNA-TARE-4L (***). Positive probe hybridization intensities are plotted from each sample after quantile normalization, log2 transformation, and median centering.

Our transcriptional profiling approach identified 1,360 significantly expressed TARs. Specifically, 1,229 TARs exhibited overlap with 872 probed *PlasmoDB v6.5 *protein-coding genes, 8 TARs overlapped the 2 probed ribosomal RNA genes, and 123 TARs represented un-annotated, putative non-coding transcripts from *P. falciparum *intergenic regions (Figure 1a). In summary, both ribosomal RNA genes and 64.1% of protein-coding genes on the array were identified as expressed in at least one of the four time-points tested. This is consistent with previous studies showing that 70 to 90% of protein-coding genes are expressed during *P. falciparum *intraerythrocytic development [3-5]. Coordinates for all predicted TARs are included in Additional file 2.

We next investigated our expression data for known patterns of gene regulation. We performed clustering analysis to confirm the stage-specific transcription cascade expected for *P. falciparum'*s protein-coding genome (Figure 1b; Additional file 3), and ontology analysis of stage-specific genes to confirm the parasite processes known to occur during our time-points (Additional file 4). As a further control, we confirmed that known housekeeping genes such as seryl-tRNA synthetase (PF07_0073) were not differentially expressed, while early and late stage markers such as PFD0020c and PFB0275w were maximally expressed in T18 and T36, respectively (Figure 1c) [3-5]. Collectively, these analyses confirmed the biological representation of our samples and provided meaningful context to investigate lncRNA expression during the parasite's pathogenic human blood stage.

We applied conservative criteria to identify *bona fide P. falciparum *lncRNAs from the set of 123 expressed intergenic transcripts. Namely, we required transcripts to be larger than 200 nucleotides, eliminating 46 putative small RNAs from further analysis (Additional file 5). We also eliminated transcripts having even marginal protein-coding potential. Briefly, we used BLASTX to translate the remaining 77 transcripts and search for significant protein matches across all 439,884 and 12,597,337 sequences represented in the Swissprot and non-redundant protein sequence (nr) databases, respectively. We also searched subsets of both databases with the following organism queries: *Plasmodium*, *Plasmodium falciparum*, and *Plasmodium falciparum strain 3D7*. While the large majority of analyzed transcripts lacked any coding potential, BLASTX analysis predicted that 17 transcripts might, in fact, be novel *P. falciparum *genes or pseudogenes (Additional file 6). Thus, our conservatively filtered set of putative lncRNAs for additional characterization consisted of 60 candidate sequences (Figure 1a; Additional file 7).

### Additional characterization of lncRNA candidates highlights a novel telomere-associated lncRNA

To systematically prioritize the 60 putative lncRNAs for functional follow-up, we looked for lncRNAs with similar properties to known functional transcripts in *P. falciparum *and/or *bona fide *lncRNAs in other organisms. We also investigated the possibility that lncRNA candidates may be spliced to nearby genes or represent un-annotated UTRs. To this end, we assessed each lncRNA candidate's G+C content and evolutionary conservation, as well as the correlation between lncRNA and neighboring gene expression profiles, and the distance between lncRNA and neighboring genes to infer transcript independence.

**Figure 2:**
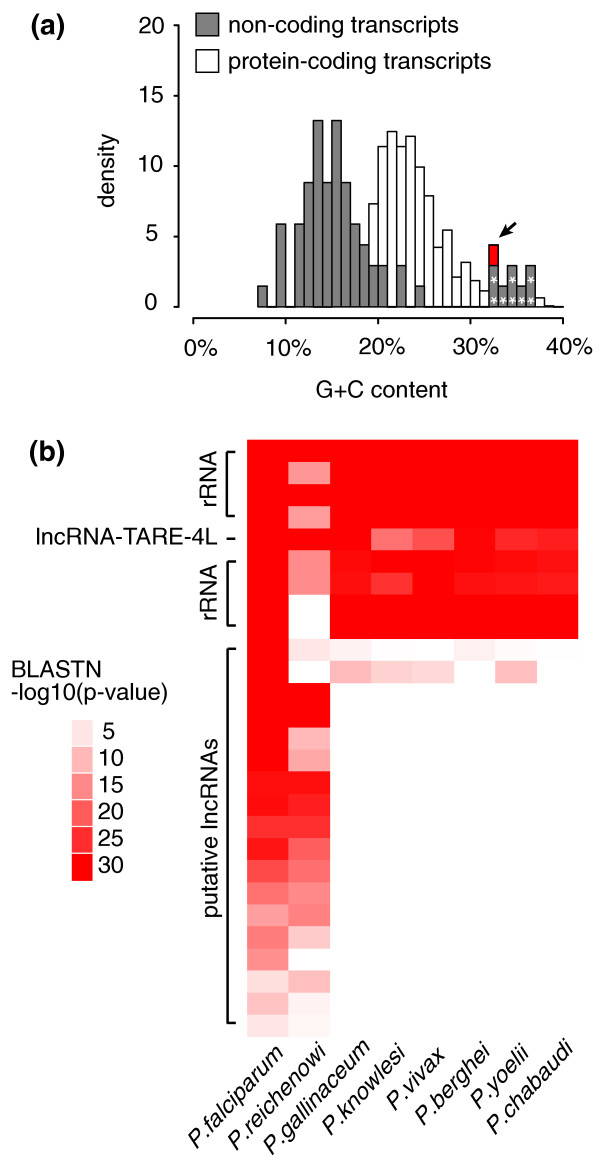
**Additional characterization of putative lncRNAs highlights the striking properties of a telomere-associated lncRNA**. **(a) **Guanine-cytosine (G+C) content comparison between predicted non-coding and protein-coding transcripts shows that non-coding G+C content is typically lower than coding transcript G+C content. lncRNA-TARE-4L (highlighted in red and indicated by arrow) and ribosomal RNAs (indicated by asterisks) have high G+C content. **(b) **The sequence of eight functional rRNAs is broadly conserved across *Plasmodium *species. The sequence of 19 putative lncRNAs is also significantly conserved in at least one other species (BLASTN *P*-value < 0.01), suggesting a preserved functional role. lncRNA-TARE-4L is strongly conserved across all eight species analyzed. Such a broad level of conservation is rarely seen in randomly sampled intergenic regions from *P. falciparum *(*P*-value = 0.045). Rows are ordered by number of species with BLASTN hits (*P*-value < 0.01), and then by strength of hits (defined by the product of *P*-values). Species are clustered based on the phylogeny proposed by Escalante and Alaya [86]. BLAST, basic local alignment and search tool.

Known classes of functional non-coding RNA in *P. falciparum*, such as ribosomal RNA and transfer RNA, have high G+C content [32,33]. While high G+C content is certainly not sufficient or necessary for function, we were nonetheless interested in the G+C content of our putative lncRNAs. We found the average G+C content of lncRNA candidates (15.4%) to be typical of *P. falciparum *intergenic regions [34] and well below the coding transcript average (23.7%). This is not an unexpected result and, importantly, indicates no systematic hybridization bias towards detection of expressed non-coding regions with higher than expected G+C content. Interestingly, however, this analysis highlighted one lncRNA with similar G+C content (32.1%) to ribosomal RNA transcripts (Figure 2a). We termed this candidate lncRNA-TARE-4L, as it is encoded in the TARE tract on the left end of chromosome four.

We next explored the evolutionary conservation of putative lncRNA sequences as an indicator of preserved functionality [35]. We performed BLASTN sequence alignment within the partially assembled, closely related *Plasmodium reichenowi *chimpanzee parasite genome and across six other partially sequenced, more distant *Plasmodium *species (Figure 2b). We found that 19 lncRNAs exhibited some level of conservation in the *Plasmodium *species investigated, but that only lncRNA-TARE-4L was strongly conserved across all species tested. To assess the significance of this result, we repeated this analysis for 600 size-matched random intergenic sequences, as well as for ribosomal RNAs and size-matched coding exons. We found that, respectively, 4.5%, 87.5%, and 33.3% had BLASTN hits to all *Plasmodium *species analyzed. Thus, the broad sequence conservation of lncRNA-TARE-4L is significant (*P*-value = 0.045) and more similar to the level of conservation expected for ribosomal RNAs and coding regions. Interestingly, we also found that 23 of the 27 broadly conserved null intergenic sequences map to either the telomeric or subtelomeric repeats.

The vast majority of *P. falciparum *genes are highly expressed only once per 48-hour intraerythrocytic developmental cycle, and genes in related cellular processes are induced together [3]. Given this model for functional protein-coding transcripts, we reasoned that lncRNA candidates are more likely biologically significant if expressed in a stage-specific manner. Moreover, regulatory lncRNAs have been reported to act in *cis *to their targeted loci [35-37]. Consistent with this notion, lncRNAs that are both differentially expressed and encoded nearby essential or pathogenic genes may be involved in regulating these important loci. Figure 1d,e shows that putative lncRNAs are developmentally regulated similar to protein-coding transcripts (see Materials and methods and Additional file 3 for detailed comparisons). Additionally, we found that many lncRNA candidates neighbor essential genes and factors involved in parasite pathogenesis (Additional file 8).

Given clear patterns of transcriptional regulation, we next tested if the 60 candidate lncRNAs were likely to be independent transcripts as opposed to un-annotated UTRs or small spliced exons to neighboring coding genes. To this end, we measured the Pearson correlation between putative lncRNA and neighboring gene expression profiles, conservatively selecting the most correlated, adjacent, expressed gene as the neighboring gene (Additional file 7, columns j and l; and see Materials and methods). We found that 40 lncRNA candidates were highly correlated, raising the possibility that they may be spliced to or represent UTRs of adjacent coding genes. Of the remaining 20 lncRNA candidates exhibiting patterns of correlation consistent with independent transcription, 16 displayed biologically meaningful variation across our time-points. Thus, correlation analysis highlighted 16 candidates with clear evidence of independent transcriptional regulation.

While the null distribution of correlations from adjacent pairs of coding genes demonstrated that approximately 30 of 40 highly correlated lncRNA candidates may be UTRs or otherwise spliced to nearby genes, it also suggested that 10 independent transcripts should exhibit this level of correlation by chance (Additional file 9). To further distinguish such potential transcripts, we considered the distance of each putative lncRNA to the nearest neighboring coding gene. We found that 11 lncRNA candidates were over 1 kb from a coding gene. As the mean intron length in *P. falciparum *is 168 bp [34], we considered this as evidence of independence regardless of correlation value. Taken together, these results point to a highly conservative set of 23 lncRNA candidates based on correlation and distance analyses.

**Figure 3:**
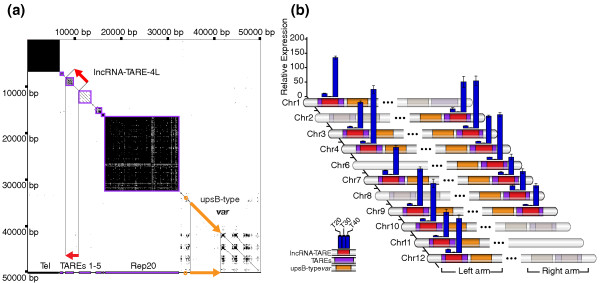
**The telomere-associated lncRNA-TARE family neighbors upsB-type *var *loci and is coordinately expressed during parasite schizogony**. **(a) **Dot plot analysis of the first 50,000 bp on chromosome 4 pinpoints lncRNA-TARE-4L in the TARE 2-3 region between the telomere (Tel) and a silenced upsB-type *var *virulence gene. **(b) **Subtelomeric features are diagrammed on the 11 chromosomes (Chr) from which we were able to specifically amplify lncRNA-TARE sequence. Features are not to scale, and grayed out chromosome ends were not probed. Notably, an upsB-type *var *gene (orange) is located adjacent to each predicted lncRNA-TARE gene (red). Furthermore, qRT-PCR amplification of 15 distinct lncRNA-TARE genes shows a coordinated pattern of late stage expression. lncRNA-TARE relative expression is plotted in time-points T20, T30, and T40 ± 3 hpi with respect to T30. Error bars represent the propagated standard error of the mean (pSEM) from two biological replicate experiments. Housekeeping gene PF08_0085 was used as the reference gene.

Strikingly, we found that lncRNA-TARE-4L exhibited the strongest evidence of independent transcriptional regulation. lncRNA-TARE-4L is flanked by the telomere and an upsB-type *var *gene (Figure 3a). However, the *var *gene is over 20 kb away and the exon structure of *var *transcripts has been previously mapped [38,39]. Given a mean intron length of 168 bp and maximum intron length of 4.9 kb in *P. falciparum *[34], the splicing of lncRNA-TARE-4L to the nearest neighboring *var *gene would be biologically unprecedented. Moreover, correlation analysis revealed that lncRNA-TARE-4L is anti-correlated with the nearest expressed gene (r = -0.296). The nearest expressed gene (PFD0020c) is an additional 20 kb beyond the silenced upsB-type *var *gene and is profiled in Figure 1c for visual comparison to lncRNA-TARE-4L (Figure 1e, asterisks).

Collectively, lncRNA-TARE-4L emerged as the lncRNA candidate with the most promising properties for functional follow-up. lncRNA-TARE-4L is encoded in the TARE 2-3 subtelomeric repeat region between the telomere and a silenced upsB-type *var *gene (Figure 3a), has G+C content and sequence conservation similar to that of functional ribosomal RNA, is sharply induced after parasite DNA replication, and is clearly an independent transcript.

As we further investigated the sequence properties of lncRNA-TARE-4L, we found that homologous lncRNA-TARE sequences are encoded adjacent to upsB-type *var *genes on 22 of 28 *P. falciparum *chromosome ends (Figure 3b) [40-42]. In five cases where there is no lncRNA-TARE gene, there is similarly not an upsB-type *var *gene. This perhaps suggests concurrent evolutionary pressure acting on lncRNA-TARE and upsB-type *var *genes. Consistent with this notion, the lncRNA-TARE loci are highly similar; the average pairwise identity between sequences is 88.1% (Additional file 10).

### lncRNA-TARE loci are coordinately expressed from at least 15 chromosome ends

Given our evidence for expression of the TARE 2-3 region on chromosome 4 and conservation of this region on 22 *P. falciparum *chromosome ends, we hypothesized that the 22 TARE 2-3 regions may be coordinately expressed. We thus set out to further investigate the expression properties of lncRNA-TARE loci in independent blood stage time courses. In line with our array results, we expected lncRNA-TARE-4L, and potentially other lncRNA-TARE genes, to be differentially expressed after parasite DNA replication.

We conducted two additional highly synchronous *P. falciparum *time courses focused on stage-specific time-points T20 (ring), T30 (trophozoite), and T36/T38/T40 (schizont) ± 3 hpi, and subsequently isolated RNA from each stage. We were able to design specific primer pairs targeting 15 chromosome ends and used qRT-PCR to probe expression at the TARE 2-3 region. Primer pairs were excluded if they did not have at least 90% amplification efficiency or amplified non-specific products (Materials and methods; Additional file 11).

We found that the TARE 2-3 region is expressed on all 15 distinct chromosome ends tested. Remarkably, all 15 lncRNA-TARE genes are coordinately and strongly induced after parasite DNA replication, with maximal lncRNA-TARE transcript abundance observed in the T40 ± 3 hpi time-point (Figure 3b). This result is the first quantitative experimental evidence showing that 15, if not more, *P. falciparum *chromosome ends are transcriptionally active between the parasite's DNA replication and cell division cycles. Taken together, we have validated and expanded the microarray discovery of lncRNA-TARE-4L to define a novel telomere-associated lncRNA family, termed lncRNA-TARE. We have also shown that lncRNA-TARE is maximally expressed during an important stage of parasite blood stage development.

### RACE defines two dominant long telomere-associated transcripts

**Figure 4:**
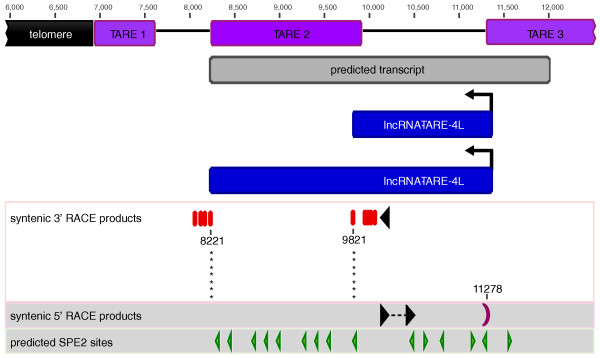
**RACE suggests two long telomere-associated transcripts are transcribed in the TARE 2-3 region**. 3' RACE products of seven distinct lncRNA-TARE sequences terminated at a precise syntenic site on either side of TARE 2 (asterisks). Taken together with the 5' RACE product suggests two species approximately 1.5 kb and 3.1 kb in length are transcribed towards the telomeres from the TARE 3 boundary. Genome-wide, the palindromic SPE2 DNA motif (green triangles) is enriched only in the TARE 2-3 region from which lncRNA-TARE is transcribed and in the upstream promoter element of upsB-type *var *genes. Primers are depicted as black triangles and numbers represent base-pair coordinates corresponding to the left end of chromosome 4. SPE2, subtelomeric *var *gene promoter element 2.

We next pursued RACE to map putative transcriptional start and termination sites of lncRNA-TARE. We used a priming strategy in which both the 5' and 3' RACE reactions were primed using the same primer sequence, albeit reverse complemented, to ensure amplification of a contiguous long transcript. Additionally, as we aimed to investigate the transcript boundaries of multiple lncRNA-TARE loci in parallel, we designed RACE primers to simultaneously target 20 lncRNA-TARE sequences (Figure 4, black triangles; Additional file 11).

3' RACE analysis suggested two dominant lncRNA-TARE species are transcribed from the centromere towards the telomere on at least 12 different chromosome ends (Figure 4; Additional files 12 and 13). Interestingly, we found that the longer transcript corresponded to termination immediately after the TARE 2 element, while the shorter transcript corresponded to termination immediately prior to TARE 2. Additionally, the syntenic termination site for seven distinct lncRNA-TARE family members encompassing TARE 2 was identical and corresponded to the transcript model predicted by our sliding-window algorithm (Materials and methods). In terms of the shorter transcript, we again found that seven distinct lncRNA-TARE species shared a precise syntenic termination site just upstream of TARE 2 (Figure 4, asterisks).

We discovered a single putative 5' transcriptional start site for the lncRNA-TARE locus on the left end of chromosome 3. Notably, this start site corresponded to the boundary of the TARE 3 element (Figure 4; Additional files 12 and 13). The intrinsic tendency of PCR amplification of multi-gene families to result in strongly biased representation of certain family members [43] likely explains the homogeneity of our cloned 5' RACE products.

Collectively, our RACE results provide strong evidence that long non-coding RNA genes are present in the *P. falciparum *TARE 2-3 subtelomeric repeats. Furthermore, our results support a model of unidirectional transcription towards the telomeres, as we were unable to amplify transcripts of the opposite polarity. We note, however, that our results do not explicitly rule out bidirectional transcription or the presence of alternative transcript models beyond the approximately 1.5 kb and 3.1 kb species defined here. For example, the high A+T content of the *P. falciparum *genome poses a technical barrier for 3' RACE analysis, as internal A-rich regions may hybridize with the 3' RACE primer targeted to poly-A transcript tails.

### lncRNA-TARE loci are enriched with transcription factor binding sites

An additional salient feature of lncRNA-TARE is that the TARE 2-3 region contains approximately 15 occurrences of the bipartite, palindromic subtelomeric *var *promoter element 2 (SPE2) motif on average [44]. This pattern seems to be non-random as there are only two SPE2-enriched loci along the *P. falciparum *genome: the TARE 2-3 region and the upstream promoter element of subtelomeric upsB-type *var *genes. Notably, these two loci account for 94% of 777 predicted SPE2 consensus sites, and both the presence and position of SPE2 sites is conserved on intact chromosome ends (Figure 4; Additional file 10) [10,42,44,45].

Moreover, Flueck *et al*. [44] and others [10,46,47] have recently demonstrated that a member of *P. falciparum*'s ApiAP2 transcription factor family specifically binds subtelomeric SPE2 sites in late stage parasites. We thus further investigated, using qRT-PCR, the expression of lncRNA-TARE-4L and the *P. falciparum *SPE2-interacting protein (PfSip2; PFF0200c). We compared expression at T30 ± 3 hpi during peak DNA replication to expression at T36, T38, and T40 ± 3 hpi during parasite schizogony. We found lncRNA-TARE-4L and PfSip2 to have highly correlated late stage temporal profiles. Our results also suggested that PfSip2 is expressed prior to maximal lncRNA-TARE-4L expression, which may indicate PfSip2 induction of the lncRNA-TARE locus and/or co-activation of lncRNA-TARE (Additional file 14).

## Discussion

**Figure 5:**
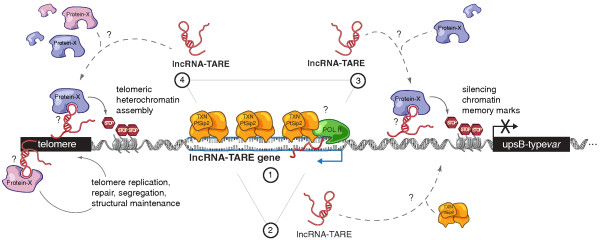
**Potential models for lncRNA-TARE at *P. falciparum *chromosome ends**. Model 1: PfSip2 binding at SPE2 sites induces lncRNA-TARE transcription and/or vice versa. Model 2: lncRNA-TARE guides multiple copies of PfSip2 to chromosome ends. Model 3: lncRNA-TARE recruits chromatin-remodeling factors to initiate post-S-phase silencing chromatin memory marks at upsB-type *var *loci. Model 4: lncRNA-TARE facilitates telomeric heterochromatin assembly and/or interacts with telomere-associated proteins. POL II, RNA polymerase II; TXN, transcription factor.

In the present work we have identified and characterized several previously undetected lncRNAs in *P. falciparum*, such as a novel family of 22 homologous lncRNA-TARE genes that exhibit coordinated expression at a key stage in the *P. falciparum *life cycle. This family of lncRNA-TAREs encompasses the majority of known binding sites (SPE2) for the ApiAP2 transcription factor PfSip2 (Figure 4; Additional file 10). As PfSip2 is expressed and specifically binds subtelomeric SPE2 sites at the stage of maximal lncRNA-TARE transcription, these results suggest that PfSip2 binding may positively regulate the lncRNA-TARE locus [44]. Alternatively, lncRNA-TARE transcription may allow PfSip2 binding, or in the case that both models are valid, a regulatory feedback loop between PfSip2 binding and lncRNA-TARE transcription may exist (Model 1 in Figure 5) [48].

The only other conserved cluster of SPE2 sites is in the upstream promoter element of upsB-type *var *genes. This suggests that lncRNA-TARE may play a role in subtelomeric upsB-type *var *gene regulation. Consistent with this notion, all 22 lncRNA-TARE genes are encoded adjacent to an upsB-type *var *gene. Moreover, PfSip2 is implicated in silenced subtelomeric *var *gene regulation [44]. Thus, lncRNA-TARE may help regulate upsB-type *var *genes by directly or indirectly interacting with and/or recruiting multiple copies of PfSip2 to chromosome ends (Model 2 in Figure 5) [49].

Mutually exclusive *var *gene regulation is known to involve nuclear repositioning and heritable chromatin memory marks [6,50-55]. An emerging theme in mammalian epigenetic regulation is the association of lncRNAs with chromatin modifying complexes, which in turn recruits these factors to their sites of action [36,56-58]. Notably, the induction of lncRNA-TARE transcription between *P. falciparum *DNA replication and cell division is ideally timed to help initiate post-S-phase epigenetic memory marks at nearby upsB-type *var *genes. Thus, it is possible that lncRNA-TARE may interface with chromatin reading and/or writing factors to modulate the precise epigenetic regulation of nearby subtelomeric *var *loci (Model 3 in Figure 5).

Non-coding transcripts expressed from a conserved bidirectional promoter in *var *gene introns provide some precedent for the proposed link between lncRNA-TARE and *var *gene regulation. Perhaps non-coincidentally, both lncRNA-TARE and *var *gene 'sterile' transcripts have a similar post-S-phase transcriptional profile [59]. However, while the 22 lncRNA-TARE loci exclusively pair with upsB-type *var *genes, the previously described *var *gene 'sterile' transcripts are expressed from both silenced and activated *var *genes of all types [38,60]. Thus, lncRNA-TARE may provide a different and/or additional level of regulation unique to subtelomeric upsB-type *var *loci. We also note that in a complementary study by Raabe *et al*. [26], small subtelomeric non-coding RNAs ranging in size from 31 to 103 nucleotides were identified in this region. Taken together, our results perhaps support a model of long precursor transcripts being processed into small non-coding RNAs.

Interestingly, the origin and transcriptional profile of lncRNA-TARE is strongly reminiscent of the eukaryotic non-coding RNA family TERRA. Across numerous eukaryotic model systems, TERRA is transcribed from subtelomeric loci, associates with telomeric repeats, and is implicated in telomere structural integrity and heterochromatin assembly [20-25,61-66]. Consistent with a possible TERRA-like role for lncRNA-TARE, large chromatin state changes occur at *P. falciparum *chromosome ends in concordance with maximal lncRNA-TARE transcription (Model 4 in Figure 5) [67,68]. Moreover, TERRA transcript levels are regulated by the cell cycle, with lowest TERRA abundance observed during DNA replication [22,69]. We similarly observe the lowest lncRNA-TARE transcript levels at T30 ± 3 hpi, which corresponds to peak parasite DNA replication.

In summary, several pieces of evidence point to a key role for lncRNA-TARE in transcriptional and/or epigenetic regulation of *P. falciparum *telomeric and subtelomeric regions. This work motivates further experimentation to resolve the mechanistic details of lncRNA-TARE and other promising *P. falciparum *lncRNA candidates.

## Conclusions

Our global transcriptional profiling study provides a catalyst for in-depth functional analyses of high-confidence *P. falciparum *lncRNA candidates and for a full-genome investigation of parasite strains beyond 3D7. We have identified and characterized 60 putative lncRNAs using conservative thresholds for statistical and biological significance, providing 23 strong candidates for further functional experimentation, such as RNA binding assays and expression knockdown experiments. Such future studies will be key in establishing a direct molecular link between specific lncRNA transcripts and parasite proteins and in determining genes modulated by lncRNAs during *P. falciparum *blood stage development. Recent studies in model eukaryotes suggest that lncRNAs represent a new paradigm in genome regulation and chromatin remodeling. Hence, profiling the non-coding transcriptome of drug-resistant parasites, parasites with mis-regulated virulence gene phenotypes, and hyper-virulent clinical isolates is an exciting new research direction in the quest to eradicate malarial disease.

## Materials and methods

### Microarray design

The DNA tiling array was designed in conjunction with Roche NimbleGen to tile a portion of the *P. falciparum *genome with a targeted median probe spacing of 12 bp. The 3D7 assembly (PlasmoDB v5.5) was used as the reference sequence. The design targeted all of chromosomes 2, 4, 7 and 9 and partial chromosomes 3:106138-147339, 5:947885-end, and 12:start-66805 (Additional file 1). The three chromosomes tiled in their entirety were selected at random. However, the four partially tiled chromosomes represent regions of particular interest given our hypothesis that lncRNAs may be involved in chromatin remodeling and clinically important parasite processes. Probes were variable length T_m_-matched long oligonucleotides, averaging 55 bp each. Probe sequences were screened for excessive cross hybridization to 3D7 sequence: any probes with more than five close Sequence Search and Alignment by Hashing Algorithm (SSAHA) matches were eliminated [70,71]. The final design filled 366,479 probes on the array, 96.81% of which are unique. The raw and normalized data discussed in this publication have been deposited in NCBI's Gene Expression Omnibus and are accessible through accession [GEO:GSE27937].

### Parasite culture, RNA preparation, and cDNA labeling for microarray hybridizations

A clone of *P. falciparum *strain 3D7 was cultured using standard methods [72,73] and total RNA isolated from each sample as described [74]. Total RNA was cleaned up with an RNeasy column (Qiagen, Valencia, CA, USA) and concentrated in a Microcon YM-30 centrifugal filter (Millipore, Billerica, MA, USA). Total RNA (1 μg) was then subjected to poly-A selective amplification using Message Amp II (Ambion, Foster City, CA, USA), substituting biased dNTP/NTP mixes (2A/T/U:1C/G) for the solutions provided. The resulting aRNA was labeled with Superscript II reverse transcriptase (Invitrogen, Carlsbad, CA, USA) using random hexamers, and either Cy3- or Cy5-dUTPs (GE Healthcare, Piscataway, NJ, USA) for 2 hours at 42°C following a 10-minute primer annealing step at 65°C. The reaction was concentrated on a Microcon YM-30 column and subjected to array hybridization per standard NimbleGen protocol.

### Data normalization and quality control

Raw data from each sample was quantile normalized [75] and log2 transformed prior to prediction of TARs along the *P. falciparum *genome. We based our pre-processing pipeline on established quality control metrics: removal of non-biological variation and a strong correlation between raw and normalized data [76]. Additional file 15 shows log2(intensity) distributions of each sample before and after quantile normalization, log2(intensity) boxplots of each sample after quantile normalization, and all pair-wise correlation scatterplots of data before and after normalization. Pearson correlation is equal to 1.0 between raw and normalized data in all matched samples. Additional file 16 shows that, for each sample, we observed only a minimal increase in median intergenic probe hybridization intensity with number of G+C bases. Given that we make no absolute or quantitative expression comparisons of transcripts (only relative expression comparisons of the same transcript across time-points) and we confirm no G+C content bias in predicted lncRNAs, we deemed this inconsequential. Normalized data were median centered (at zero) prior to expression profiling and data browsing in Integrated Genomics Viewer [77].

### Detection of TARs from tiling arrays

We wrote and implemented an iterative sliding window algorithm to scan each sample's normalized probe hybridization intensity values for statistically significant TARs. Specifically, we used a single-step maxT permutation procedure (1,000 permutations) to transform the mean probe intensity score 'T' calculated in each of the approximately 366,000 possible window slides along normalized data into a multiple-hypothesis adjusted *P*-value [29-31]. We then discarded windows with adjusted *P*-values greater than 0.05 to control the family-wise error-rate of windows predicted to be significant at 5%. We repeated this procedure a total of 28 times using window sizes of 5, 10, 15, 20, 25, 30, and 40 probes. Next, we intersected all significant windows with PlasmoDB v6.5 gene annotations, and merged overlapping annotated windows to define the boundaries of 1,229 protein-coding TARs and 8 ribosomal RNA TARs. Similarly, we merged overlapping windows that did not overlap any known or predicted gene to define the boundaries of 123 un-annotated TARs. BEDTools v4 was used for all data intersections and unions [78]. Additional file 2 lists predicted TAR coordinates.

### Filtering un-annotated TARs

We filtered un-annotated TARs by setting a minimum length criterion of 200 bp and ensuring no BLASTX predicted coding potential. Out of 123 predicted un-annotated TARs, 46 were under 200 bp in length (Additional file 5). We retrieved FASTA sequence for the remaining 77 from PlasmoDB v6.5, and used the NCBI BLASTX web server to search for any significant protein matches [79]. Default BLASTX settings (BLOSUM62, word size 3, low complexity filtering, and so on) were used except the Expect threshold for reporting match significance (that is, coding potential) was set at 0.01. Seventeen sequences with an Expect score < 0.01 were categorized as putative novel *P. falciparum *genes or pseudogenes and were excluded from further lncRNA characterization (Additional file 6). We searched both the Swissprot and Non-redundant protein sequence (nr) databases with the following organism queries: all organisms, *Plasmodium*, *Plasmodium falciparum*, and *Plasmodium falciparum *strain 3D7.

### Gene Ontology term analysis of stage-specific genes

Gene Ontology (GO) term analysis of stage-specific genes was performed using GOstat with default settings [80] and Sanger GeneDB *P. falciparum *gene annotations. Stage-specific genes were determined by intersection of PlasmoDB v6.5 gene annotations with protein-coding transcripts maximally expressed in each time-point. We looked for overrepresented GO terms in stage-specific genes versus the 1,360 protein-coding genes covered by the array. Additional file 4 lists all genes covered by the array, stage-specific genes, and the top four most overrepresented GO terms in each time-point.

### Evolutionary sequence conservation

FASTA sequence for the 60 putative lncRNAs and 8 ribosomal RNA transcripts was retrieved from the 3D7 reference sequence (PlasmoDB v7.1). We also downloaded genomic FASTA sequence from PlasmoDB v7.1 representing all eight sequenced or partially sequenced *Plasmodium *species (*P. falciparum*, *P. reichenowi*, *P. gallinaceum*, *P. knowlesi*, *P. vivax*, *P. berghei*, *P. yoelli*, and *P. chaubadi*). We searched for sequence conservation using BLASTN (WU-BLAST 2.0 MP-WashU (4 May 2006)) using the same low-complexity filtering and context parameters as the PlasmoDB v7.1 BLAST server (-filter seg -ctxfactor 2.00) and setting the Expect threshold for significance to 0.01. We recorded the lowest BLASTN *P*-value within each species (Additional file 7, columns s to z).

The broad conservation of lncRNA-TARE-4L across all eight *Plasmodium *species was determined to be significant by null permutations. We chose 600 random intergenic regions from the 3D7 reference genome (based on v.7.1 annotation). These intergenic regions were sized to match the length distribution of the 60 putative lncRNAs, and were included in the WU-BLAST search. Out of 600 random intergenic regions, we found only 27 to be conserved across all 8 species, yielding an empirical *P*-value of 0.045.

### Expression profiling

To profile each predicted TAR, we calculated its expression in each time-point as the mean hybridization intensity of probes tiling within or up to 25 bp on either side of the predicted TAR start and stop coordinates. The expression profile of each TAR was then mean centered across time-points and visualized using a non-hierarchical clustering dimension reduction algorithm. Specifically, we used non-metric multi-dimensional scaling (nMDS) as implemented in the R-project 'NeatMap' package to order rows and preserve data topology. In development and validation of the 'NeatMap' package, Rajaram and Oono [81] have similarly applied nMDS to visualize yeast cell cycle expression data. We point the reader to Figure 1d of Rajaram and Oono [81] and Figure 4 of Taguchi and Oono [82] for examples and thorough discussion of the utility of nMDS in determining relational patterns of gene expression. Notably, because nMDS is a non-linear numerical optimization technique, multiple ordinations were run to select the optimal solution.

We conducted a detailed comparison of lncRNA and protein-coding expression, finding lncRNA candidates to be expressed on par with protein-coding transcripts. Included in Additional file 3 are additional visualizations of protein-coding transcript versus putative lncRNA expression, including standard heatmaps and nMDS ordinated heatmaps without mean centering across time-points. Additional file 3 also provides a histogram of the maximum expression values for lncRNA candidates and protein-coding transcripts. Notably, we found 30 lncRNA candidates (50%) to be induced by greater than two-fold across our time course samples (Additional file 7, column k). By comparison, 309 of 1,229 protein-coding transcripts (25%) match this criterion.

### Nearest-neighboring genes

Nearest neighboring genes to the set of 60 putative lncRNAs were extracted using the Cistrome Analysis Pipeline and PlasmoDB v6.5 gene coordinates.

### Correlation analysis

To infer putative lncRNA splicing or UTR relationships with neighboring coding genes, we measured the Pearson correlation between putative lncRNA and neighboring coding gene expression profiles (Additional file 7, column j). We conservatively defined the neighboring coding gene to be the highest correlated, expressed gene to either side of each putative lncRNA locus. We also examined a null distribution of correlations from adjacent pairs of coding genes. We found the 60 candidate lncRNAs to be enriched for high correlation to neighboring genes. 40 of these candidates were highly correlated (r > 0.9), whereas only 10 should be highly correlated as demonstrated by our null distribution (Additional file 9). We then further investigated the expression profiles of lncRNA candidates with r < 0.9 to ensure correlation values reflected biologically meaningful variation. We defined biologically meaningful variation as a greater than 0.5-fold change across time-points.

### Mapping of homologous lncRNA-TARE sequences

We used the PlasmoDB v6.5 BLASTN web server to record coordinates for homologous lncRNA-TARE sequences based on the predicted lncRNA-TARE-4L sequence. We then retrieved FASTA sequence of the most telomere-proximal 50,000 bp on each chromosome end from PlasmoDB v6.5, and used JDotter [83] software to create DNA dotplots mapping the telomeric repeats, TAREs 1 through 5, Rep20, and the first predicted gene on each end. We placed each predicted lncRNA-TARE gene onto the dotplot maps to confirm that lncRNA-TARE maps to TARE 2 and the sequence between TARE 2 and TARE 3 on 22 chromosome ends. We then used Geneious to cluster (ClustalW) and investigate the conservation of lncRNA-TARE sequences.

### Parasite culture for qRT-PCR analysis

Two independent biological replicate time courses were performed to validate and investigate lncRNA-TARE expression in more detail. For each time course, a freshly thawed *P. falciparum *strain 3D7 clone was cultured using standard methods [72] in human red blood cells at 4% hematocrit. RPMI-HEPES medium was supplemented with 5% human serum (O+) and 5% Albumax II (Invitrogen, Carlsbad, CA, USA). Cultures were initially synchronized using two 5% sorbitol solution treatments [73] spaced by 16 hours. To then obtain highly synchronized cultures, newly formed ring-stage parasites were selected for using 5% sorbitol solution treatments during the subsequent two re-invasion generations. Highly synchronized cultures were expanded and harvested at stage-specific time-points. Each harvested culture was centrifuged at 2,400 rpm in a Sorvall RT6000B, and packed red blood cells lysed using a 0.05% (final concentration) saponin solution. Liberated parasites were washed using phosphate-buffered saline (pH 7.4), pelleted at 13.2 rpm in a microcentrifuge, resuspended in 1 ml TRIZOL reagent, and stored at -80°C prior to RNA extraction.

### RNA preparation for qRT-PCR and RACE analysis

TRIZOL-chloroform extraction was performed and the aqueous layer applied to an RNeasy column (Qiagen). On-column DNAse digestion was carried out for 30 minutes to remove genomic DNA. Eluted RNA was also treated with TURBO DNase (Ambion) and cleaned up on a second RNeasy column (Qiagen) to yield high-purity RNA samples.

### qRT-PCR analysis

RNA (1 μg) from each time course sample was reverse transcribed using a random priming strategy (Applied Biosystems cDNA High Capacity Reverse Transcription kit; Carlsbad, CA, USA) along with a minus reverse transcriptase control reaction for each sample to confirm genomic DNA removal. qPCR reactions were carried out using 800 nM of primers and Roche FastStart SYBR Green Master mix (Indianapolis, IN, USA). Primer annealing and extension (55°C/60 seconds) was carried out for 40 cycles on an Applied Biosystems 7900 instrument.

We used PCR Miner software [84] to calculate both the cycle threshold (Ct) of each qPCR reaction and the amplification efficiency of each primer pair. We then calculated the relative expression of each lncRNA-TARE gene in each time course sample by averaging technical replicates and using the reference gene PF08_0085 and reference time-point T30 (trophozoite) for normalization. The error of normalized expression ratios was calculated using the delta method, based on a truncated Taylor series expansion, to account for technical variability in both the target and reference gene measurements. Biological replicate experiments were analyzed in isolation and then normalized expression measurements were averaged. We used a Taylor limited expansion method to determine how error propagated in the average expression value.

### qRT-PCR primer design

Primer pairs to amplify predicted lncRNA-TARE genes and the SPE2-binding protein PfSip2 (PFF0200c) were designed using Premier Biosoft International AlleleID 7.6 software (Palo Alto, CA, USA). AlleleID primer design software carries out highly specific primer design by BLAST searching sequences and masking redundant regions prior to primer design. We also independently verified primer specificity using BLASTN on the PlasmoDB v6.5 website, and ensured single amplicon melting curves and no primer dimer formation. We required primer pair amplification efficiency, as calculated by PCR Miner software [84], to be at least 90% to ensure reproducible results. We used the previously described housekeeping gene P08_0085 (ubiquitin conjugating enzyme 1) [74] to calculate all normalized relative gene expression ratios. lncRNA-TARE and PfSip2 primer sequences are listed in Additional file 11.

### Rapid amplification of cDNA ends

We employed RNA ligase-mediated RACE following manufacturer specifications (Ambion) and using 10 μg of T40 ± 3 hpi RNA mixed 1:1 from two independent time course extractions. We used Premier Biosoft International AlleleID 7.6 software to design primers targeting 20 lncRNA-TARE loci (Additional file 11). To map the putative 5' cap, we used a nested priming strategy with primers spaced roughly 350 bp antisense to the target sequence. To map 3' termini, we used a semi-nested priming strategy using a single antisense primer to the target sequence and nested primers corresponding to the 3' adapter sequence. Notably, the 5' RACE outer primer is the reverse complement of the 3' RACE primer, ensuring capture of contiguous transcripts. Minus reverse transcriptase control reactions were included for 3' RACE.

Outer and inner 5' RACE PCR cycling was performed using SuperTaq Plus polymerase (Ambion) and the following cycling conditions: 94°C for 3 minutes, 5 cycles of 94°C for 30 seconds, 60°C for 30 seconds, 68°C for 3 minutes, 35 cycles of 94°C for 30 seconds, 55°C for 30 seconds, 68°C for 3 minutes, and a final extension for 10 minutes at 68°C. 3' RACE PCR cycling was analagous except denaturation was performed at 94°C for 15 seconds and extension was performed at 68°C for 8 minutes. PCR products were gel excised, purified using Qiagen MinElute Gel Extraction Cleanup columns, and cloned into the pCR-2.1TOPO vector (Invitrogen).

We sequenced 27 and 10 colonies corresponding to 3' and 5' RACE products, respectively, using Genewiz services and Geneious analysis software [85]. A total of 12 different lncRNA-TARE loci were unambiguously represented in sequenced 3' RACE products. The original chromosome and syntenic terminus coordinates on the left end of chromosome 4 for each sequenced RACE product are included in Additional file 12 along with a graphical alignment of each sequenced RACE product to the left end of chromosome 4 in Additional file 13. RACE products were trimmed to exclude any low-quality base calls and vector sequence beyond the first four bases prior to alignment.

## Abbreviations

bp: base pair; GO: Gene Ontology; hpi: hours post-erythrocyte invasion; lncRNA: long non-coding RNA; lncRNA-TARE: long non-coding RNA telomere-associated repetitive element; lncRNA-TARE-4L: long non-coding RNA telomere-associated repetitive element on chromosome four left; nMDS: non-metric multi-dimensional scaling; PCR: polymerase chain reaction; PfSip2: *P. falciparum *SPE2-interacting protein; qRT-PCR: quantitative real-time PCR; RACE: rapid amplification of cDNA ends; SPE2: subtelomeric var gene promoter element 2; TAR: transcriptionally active region; TARE: telomere-associated repetitive element; TERRA: telomeric repeat-containing RNA; upsB: upstream sequence type B; UTR: untranslated region.

## Authors' contributions

JR and PS conceived of the study and participated in its design, coordination, and interpretation. DP carried out the computational design of arrays. JS harvested and extracted samples for arrays, and KB harvested and extracted samples for qRT-PCR and RACE. AW performed sample labeling and array hybridizations. KB analyzed raw and normalized data and wrote/implemented the sliding-window TAR detection algorithm. KB and DP analyzed predicted TARs to assess biological validity and filter for lncRNA candidates. KB and DP characterized putative lncRNAs, and KB designed/performed qRT-PCR and RACE analysis. DW, MD, SV, DVT, and UR contributed to the acquisition and interpretation of data. KB wrote the manuscript, JR and PS critically revised the manuscript, and all authors have read and given approval of the version to be published.

## Supplementary Material

Additional file 1 **DNA tiling array genome coverage**. Number of probes per 10 kb plotted by genomic position. Click here for file

Additional file 2 **All TARs**. Chromosome, coordinates, and expression profile for all 1,360 TARs identified along the *P. falciparum *genome. Click here for file

Additional file 3 **Comparison of lncRNA and protein-coding expression**. A figure providing a detailed comparison of lncRNA and protein-coding expression. Standard heatmaps and non-metric multi-dimensional scaling ordinated heatmaps of lncRNA and protein-coding transcript expression profiles without mean centering across time-points. Maximum expression value histogram for lncRNAs versus protein-coding transcripts. Click here for file

Additional file 4 **Stage-specific Gene Ontology analysis**. All genes represented on the array, stage-specific genes, and the top four over-represented Gene Ontology terms in each time-point. Click here for file

Additional file 5 **Putative small RNAs**. Chromosome, coordinates, and length of 46 putative small RNAs eliminated from the lncRNA candidate list. Click here for file

Additional file 6 **Putative genes or pseudogenes**. Chromosome, coordinates, length, and BLASTX results for 17 putative genes or pseudogenes eliminated from the lncRNA candidate list. Click here for file

Additional file 7 **Putative lncRNAs**. A table of 60 putative *P. falciparum *lncRNAs and their properties. Characteristics of 60 *P. falciparum *lncRNA candidates. Click here for file

Additional file 8 **Neighboring genes**. Nearest gene to each side of lncRNA candidates and gene descriptions. Click here for file

Additional file 9 **Correlation analysis**. Distribution of expression correlations between putative lncRNAs and neighboring coding genes as compared to the null model of adjacent pairs of coding genes. Click here for file

Additional file 10 **lncRNA-TARE homology and SPE2 sites**. A figure providing various characterizations of homologous lncRNA-TARE loci. Plot of consensus identity, location of conserved SPE2 transcription factor binding sites, and an un-rooted clustering of lncRNA-TARE loci. Click here for file

Additional file 11 **qRT-PCR and RACE primers**. A table of primer sequences used in this study. Gene-specific lncRNA-TARE/PfSip2 (PFF0200c) qRT-PCR primer sequences and family-specific lncRNA-TARE RACE primer sequences. Click here for file

Additional file 12 **RACE syntenic transcript coordinates**. Original chromosome and syntenic coordinates on the left end of chromosome 4 for each sequenced RACE product. Click here for file

Additional file 13 **RACE sequencing alignments**. Graphical alignments of each sequenced RACE product to the left end of chromosome 4. Click here for file

Additional file 14 **PfSip2 and lncRNA-TARE-4L co-expression**. qRT-PCR investigation of the lncRNA-TARE-4L versus PfSip2 locus in trophozoite and schizont stage samples. Click here for file

Additional file 15 **Raw and normalized data assessment**. Probe hybridization intensity distributions and correlation scatterplots for each sample before and after quantile normalization. Click here for file

Additional file 16 **Probe hybridization intensity versus G+C content**. Boxplots of intergenic probe hybridization intensities for each sample. Probes are grouped according to number of G+C bases. Click here for file
