# Supplementary material for: A global transcriptional analysis of Plasmodium falciparum malaria reveals a novel family of telomere-associated lncRNAs
Source: Genome Biol. 2011 Jun 20;12(6):R56. doi: 10.1186/gb-2011-12-6-r56 (PMC3218844; doi:10.1186/gb-2011-12-6-r56)
Supplement: Additional file 1 — DNA tiling array genome coverage. Number of probes per 10 kb plotted by genomic position. [file gb-2011-12-6-r56-S1.PDF]

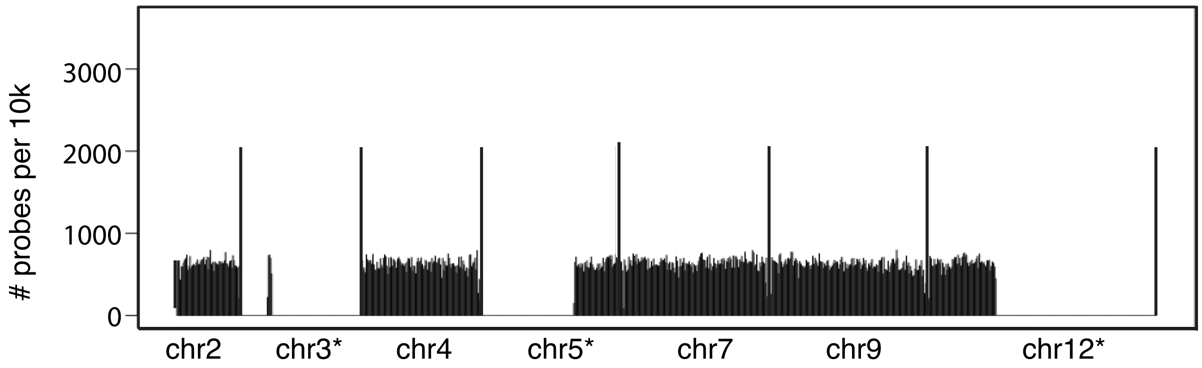

Figure A1. DNA tiling array genome coverage is dense and systematic. *P. falciparum* strain 3D7 chromosomes 2, 4, 7, and 9 are completely tiled. Chromosomes 3, 5, and 12 are partially tiled (*). Number of probes per 10000 bp is plotted versus chromosomal position. The median probe resolution is 12 bp, corresponding to approximately 830 probes per 10000 bp.
