# Supplementary material for: A global transcriptional analysis of Plasmodium falciparum malaria reveals a novel family of telomere-associated lncRNAs
Source: Genome Biol. 2011 Jun 20;12(6):R56. doi: 10.1186/gb-2011-12-6-r56 (PMC3218844; doi:10.1186/gb-2011-12-6-r56)
Supplement: Additional file 3 — Comparison of lncRNA and protein-coding expression. A figure providing a detailed comparison of lncRNA and protein-coding expression. Standard heatmaps and non-metric multi-dimensional scaling ordinated heatmaps of lncRNA and protein-coding transcript expression profiles without mean centering across time-points. Maximum expression value histogram for lncRNAs versus protein-coding transcripts. [file gb-2011-12-6-r56-S3.PDF]

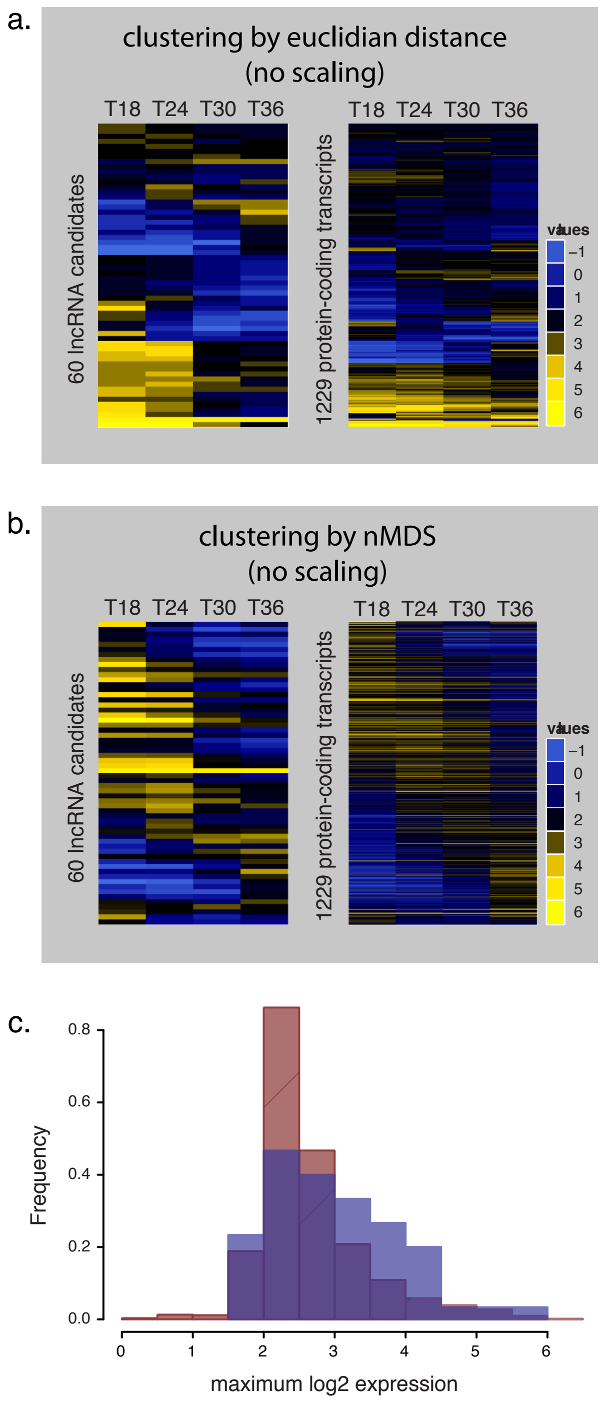

Figure A2: LncRNAs and protein-coding transcripts display similar expression. (a) hierarchical clustering of lncRNAs and protein-coding transcripts by euclidian distance without mean centering across time-points. **(b)** clustering by non-Metric Multidimensional Scaling (nMDS) without mean centering across time-points. **(c)** histogram of maximum log2 expression in any time-point for lncRNAs (blue) and protein-coding transcripts (red).
