# Supplementary material for: A global transcriptional analysis of Plasmodium falciparum malaria reveals a novel family of telomere-associated lncRNAs
Source: Genome Biol. 2011 Jun 20;12(6):R56. doi: 10.1186/gb-2011-12-6-r56 (PMC3218844; doi:10.1186/gb-2011-12-6-r56)
Supplement: Additional file 9 — Correlation analysis. Distribution of expression correlations between putative lncRNAs and neighboring coding genes as compared to the null model of adjacent pairs of coding genes. [file gb-2011-12-6-r56-S9.PDF]

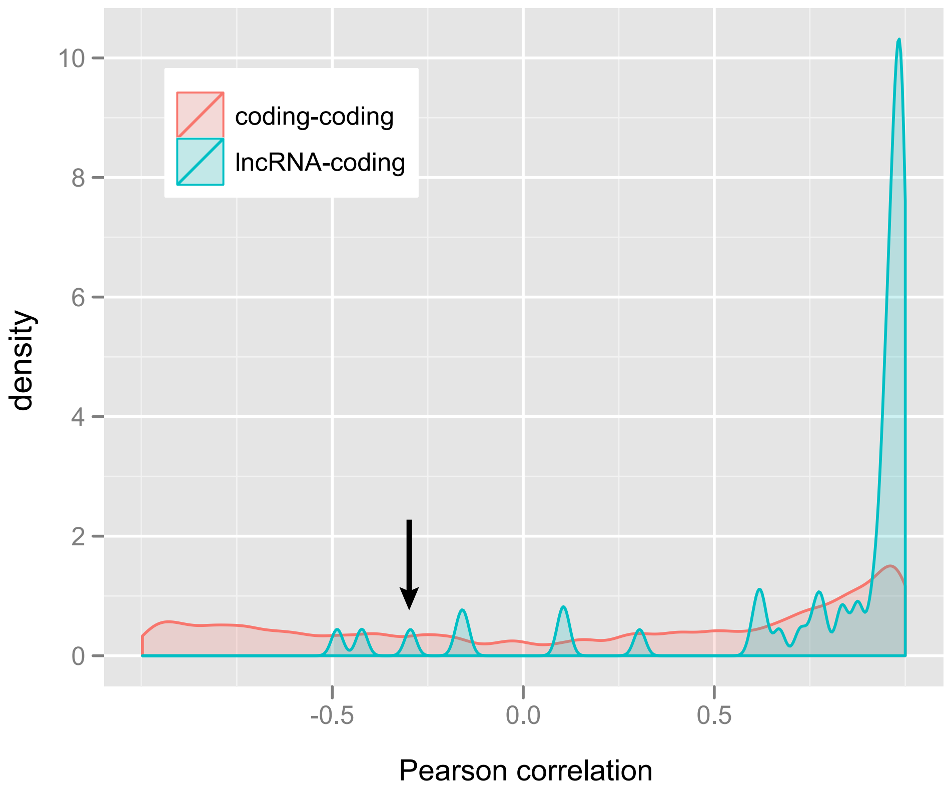

Figure A3. Distribution of time course correlations between the 60 putative lncRNAs and their neighboring coding genes (teal) versus correlations between coding genes and their neighboring coding genes (red). The set of 60 putative lncRNAs is enriched for transcripts that are highly correlated to nearby genes, suggesting that some are spliced or un-annotated untranslated regions (UTRs) to genes. The arrow marks the correlation of lncRNA-TARE-4L to its neighbor.
