# Supplementary material for: A global transcriptional analysis of Plasmodium falciparum malaria reveals a novel family of telomere-associated lncRNAs
Source: Genome Biol. 2011 Jun 20;12(6):R56. doi: 10.1186/gb-2011-12-6-r56 (PMC3218844; doi:10.1186/gb-2011-12-6-r56)
Supplement: Additional file 10 — lncRNA-TARE homology and SPE2 sites. A figure providing various characterizations of homologous lncRNA-TARE loci. Plot of consensus identity, location of conserved SPE2 transcription factor binding sites, and an un-rooted clustering of lncRNA-TARE loci. [file gb-2011-12-6-r56-S10.PDF]

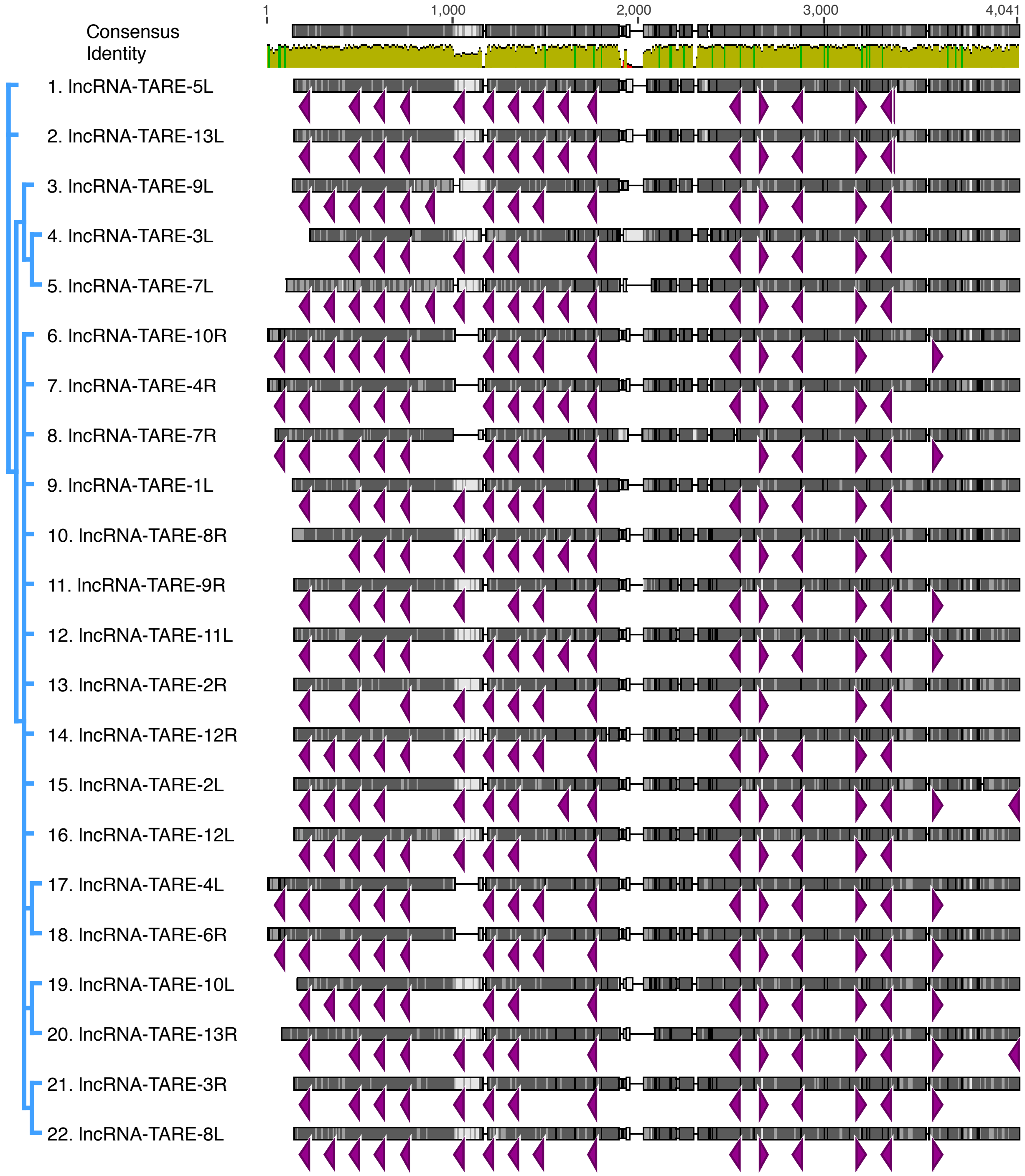

Figure A4: LncRNA-TARE homology. clustering, conservation, and SPE2 motifs within the 22 predicted lncRNA-TARE loci. Figure produced using Geneious software. Clustering performed using ClustalW.
