# Supplementary material for: A global transcriptional analysis of Plasmodium falciparum malaria reveals a novel family of telomere-associated lncRNAs
Source: Genome Biol. 2011 Jun 20;12(6):R56. doi: 10.1186/gb-2011-12-6-r56 (PMC3218844; doi:10.1186/gb-2011-12-6-r56)
Supplement: Additional file 13 — RACE sequencing alignments. Graphical alignments of each sequenced RACE product to the left end of chromosome 4. [file gb-2011-12-6-r56-S13.PDF]

a.

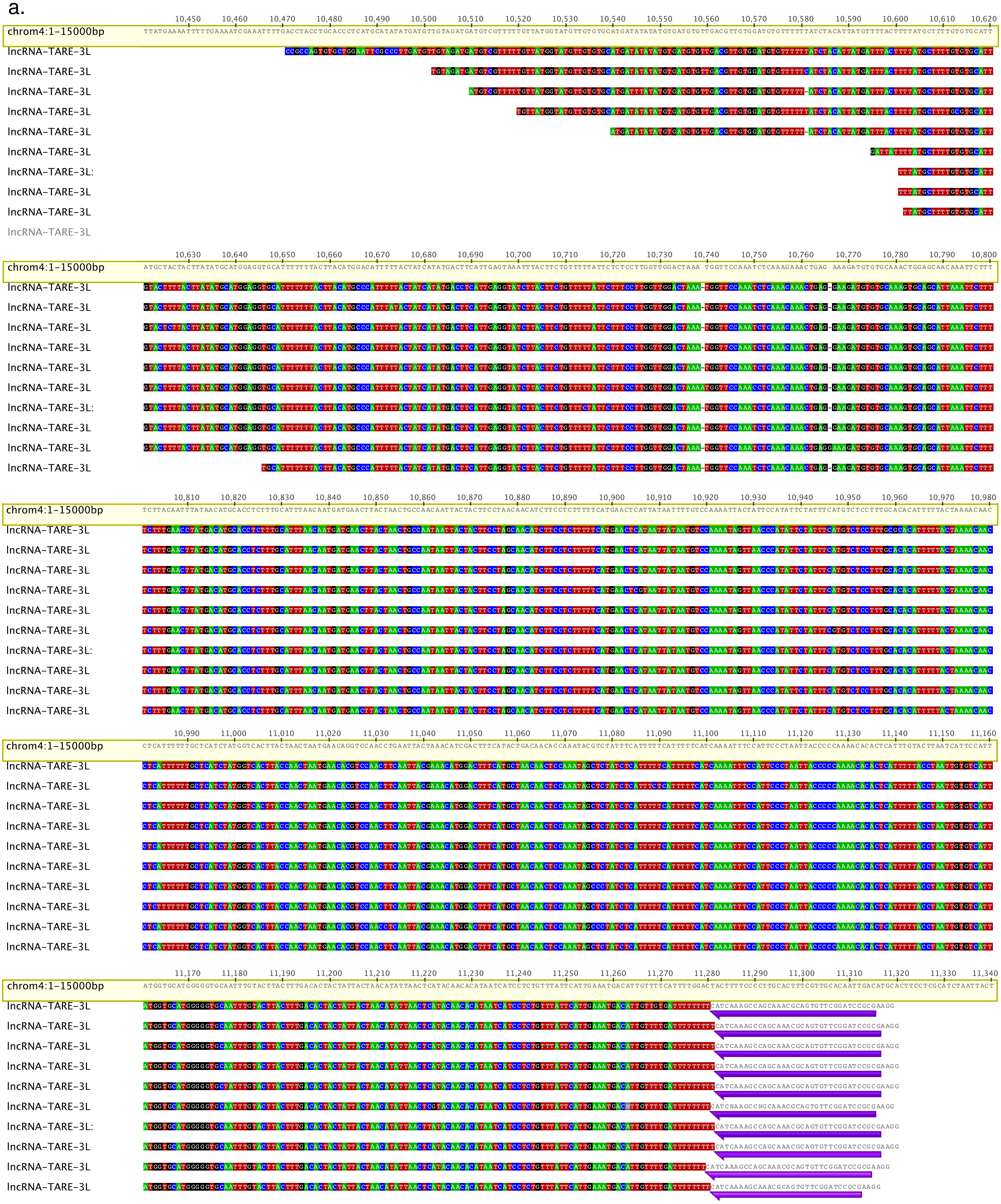

b.

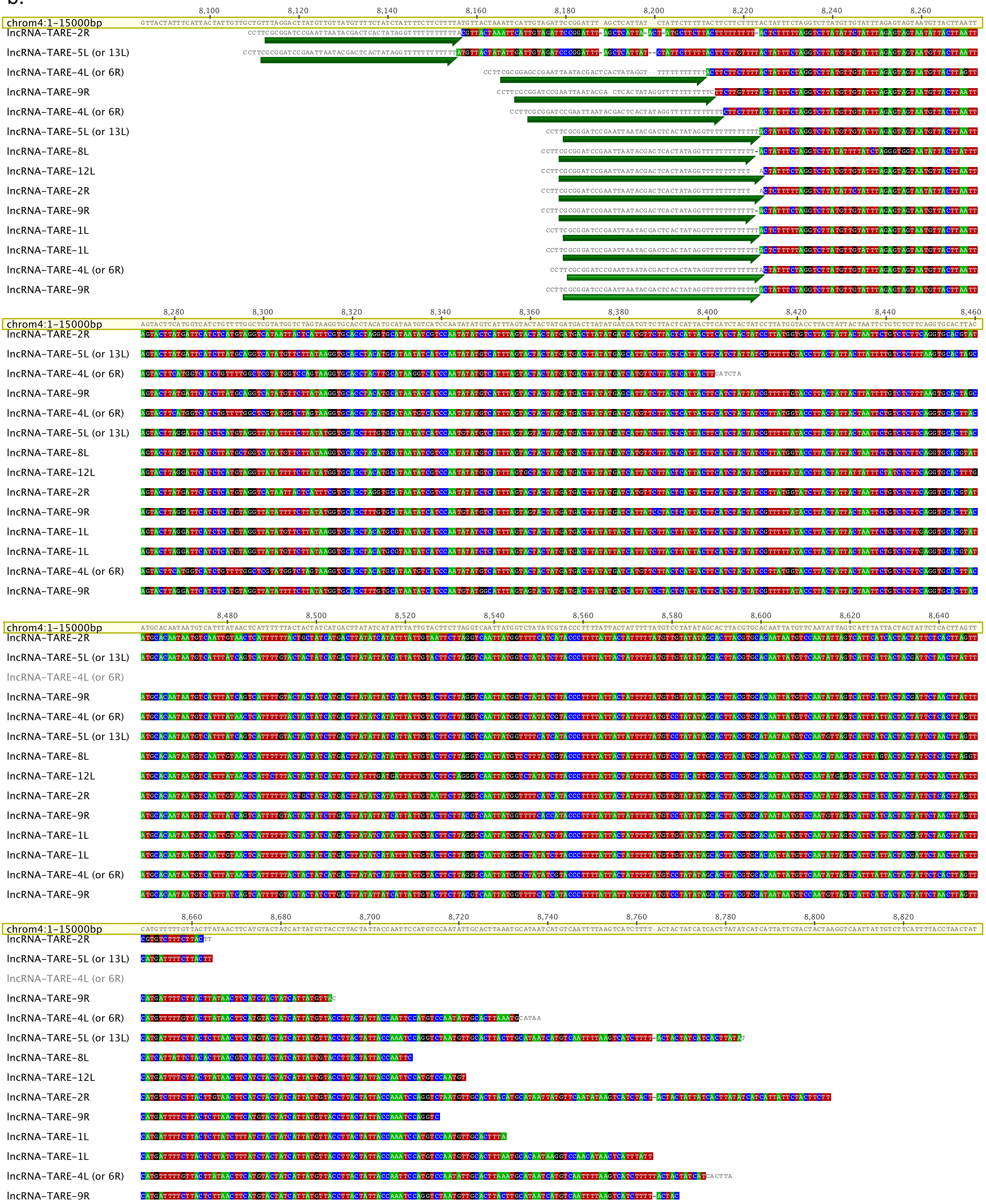

8,100 8,120 8,140 8,160 8,180 8,200 8,220 8,240 8,260
chrom4:1–15000bp
lncRNA–TARE–2R
lncRNA–TARE–5L (or 13L)
lncRNA–TARE–4L (or 6R)
lncRNA–TARE–9R
lncRNA–TARE–4L (or 6R)
lncRNA–TARE–5L (or 13L)
lncRNA–TARE–8L
lncRNA–TARE–12L
lncRNA–TARE–2R
lncRNA–TARE–9R
lncRNA–TARE–1L
lncRNA–TARE–1L
lncRNA–TARE–4L (or 6R)
lncRNA–TARE–9R
8,280 8,300 8,320 8,340 8,360 8,380 8,400 8,420 8,440 8,460
8,480 8,500 8,520 8,540 8,560 8,580 8,600 8,620 8,640
8,660 8,680 8,700 8,720 8,740 8,760 8,780 8,800 8,820

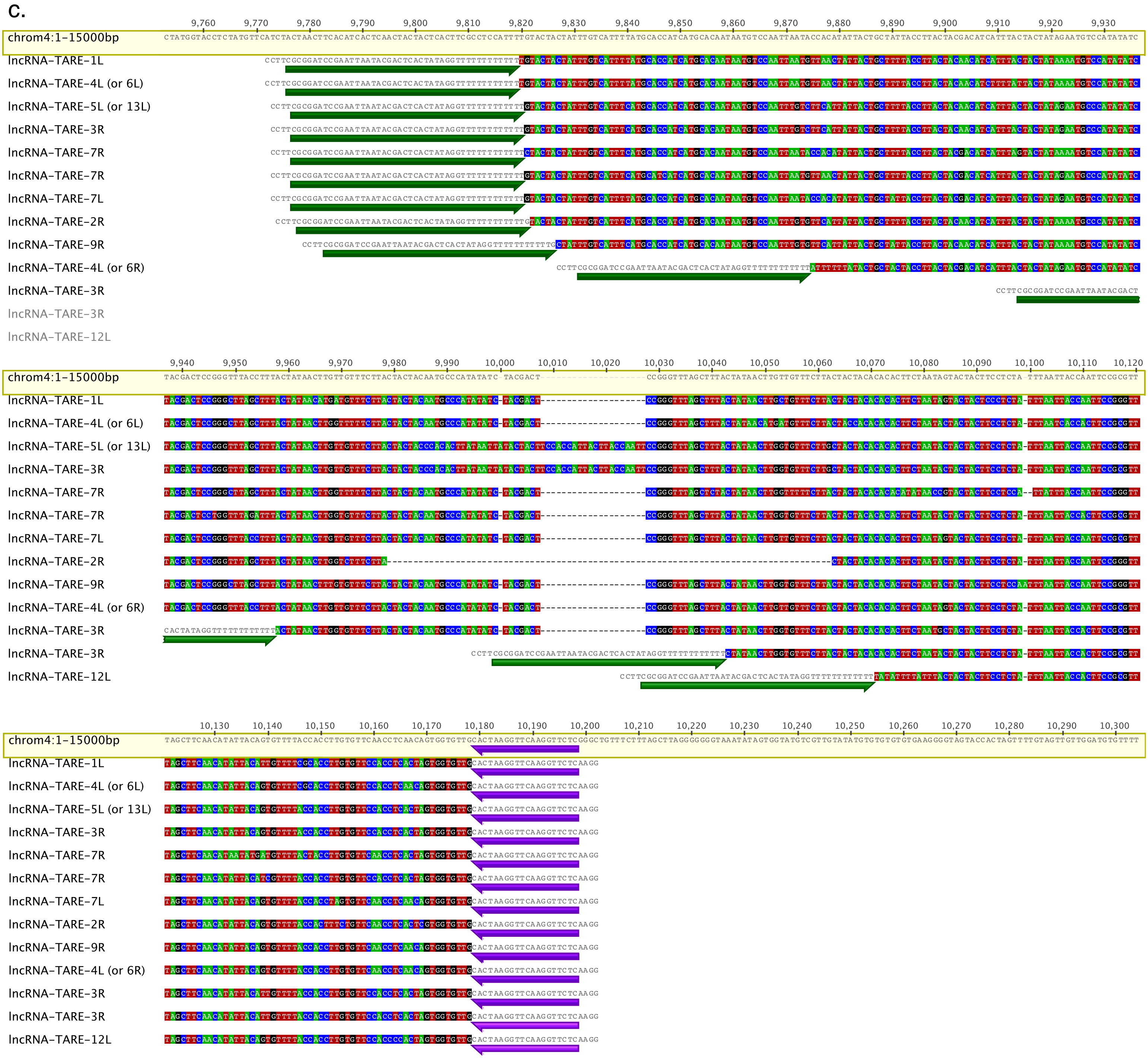

Figure A5: Sequenced lncRNA-TARE RLM-RACE products. Products were BLASTed, trimmed to exclude excessive vector sequence and low-quality base calls, and aligned to the chromosome four reference sequence using Geneious software. **(a)** 5' RLM-RACE sequencing identified a single putative transcriptional start site for lncRNA-TARE-3L corresponding to the TARE 3 subtelomeric repeat boundary. Purple arrows represent the 5' RLM-RACE adapter/primer sequence (Ambion). **(b)** 3' RACE demonstrated that a long transcript encompassing the TARE2 subtelomeric repeat is transcribed towards the telomeres. Green arrows represent the 3' RACE adapter/primer sequence (Ambion). **(c)** 3' RACE also predicted a shorter transcript terminating just upstream of TARE 2. Green arrows represent the 3' RACE adapter/primer sequence (Ambion). Purple arrows represent the lncRNA-TARE family-specific 3' RACE primer sequence. Figure produced using Geneious v4.8 software.
