# Supplementary material for: A global transcriptional analysis of Plasmodium falciparum malaria reveals a novel family of telomere-associated lncRNAs
Source: Genome Biol. 2011 Jun 20;12(6):R56. doi: 10.1186/gb-2011-12-6-r56 (PMC3218844; doi:10.1186/gb-2011-12-6-r56)
Supplement: Additional file 14 — PfSip2 and lncRNA-TARE-4L co-expression. qRT-PCR investigation of the lncRNA-TARE-4L versus PfSip2 locus in trophozoite and schizont stage samples. [file gb-2011-12-6-r56-S14.PDF]

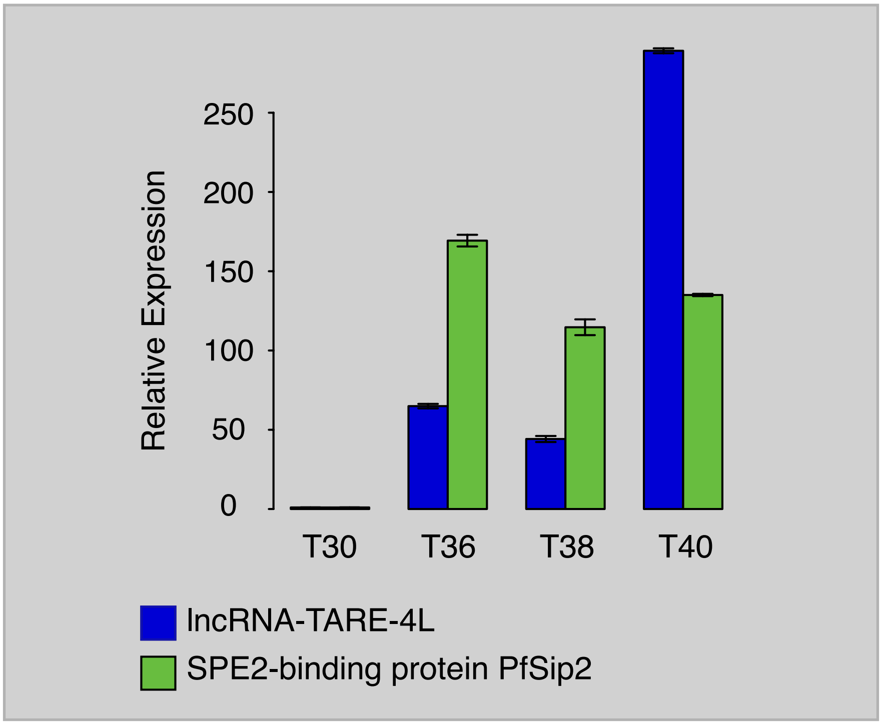

Figure A6: LncRNA-TARE is co-expressed with SPE2-binding transcription factor PfSip2. qRT-PCR amplification of lncRNA-TARE-4L and the SPE2-binding transcription factor PfSip2 demonstrates co-expression during parasite schizogony, with maximal lncRNA-TARE-4L expression occurring slightly after PfSip2 expression. LncRNA-TARE-4L and PfSip2 relative expression is plotted in time-points T30, T36, T38, and T40 +/- 3 hpi with respect to T30. Error bars represent the propagated standard error of the mean (pSEM) from four technical replicates. Housekeeping gene PF08_0085 was used as the reference gene.
