# Supplementary material for: A global transcriptional analysis of Plasmodium falciparum malaria reveals a novel family of telomere-associated lncRNAs
Source: Genome Biol. 2011 Jun 20;12(6):R56. doi: 10.1186/gb-2011-12-6-r56 (PMC3218844; doi:10.1186/gb-2011-12-6-r56)
Supplement: Additional file 15 — Raw and normalized data assessment. Probe hybridization intensity distributions and correlation scatterplots for each sample before and after quantile normalization. [file gb-2011-12-6-r56-S15.PDF]

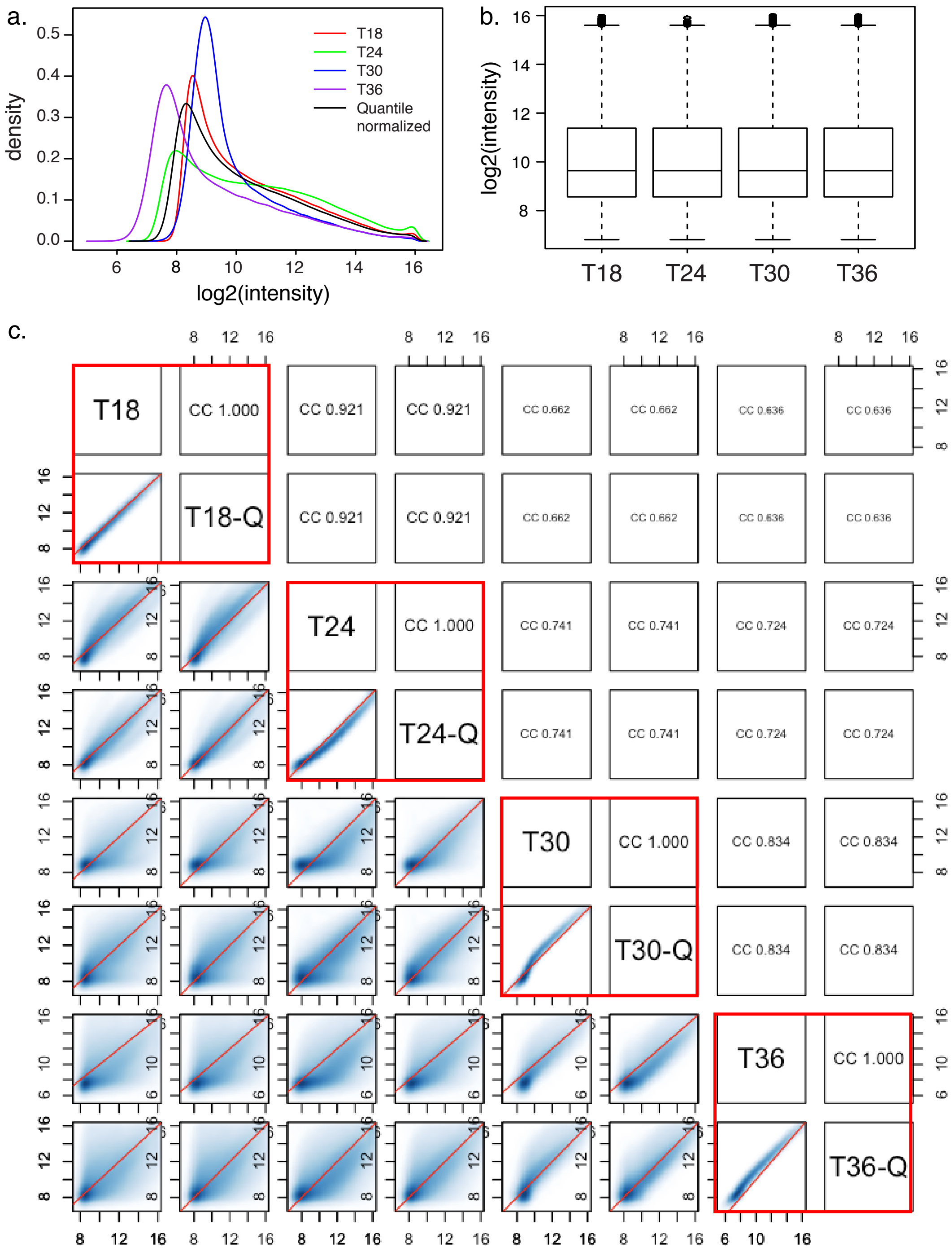

Figure A7. Quantile normalization corrects systematic biases in raw data without overcorrecting. (a) log2 probe hybridization intensity distributions from each sample prior to quantile normalization and after quantile normalization (black curve). **(b)** Boxplots of quantile normalized, log2 transformed probe hybridization intentensities from each sample. **(c)** Pairwise correlation scatterplots between raw and quantile normalized data (-Q) shows that the correlation is very strong between matched samples (Pearson's correlation coefficient (CC) = 1.0).
