# Supplementary material for: A global transcriptional analysis of Plasmodium falciparum malaria reveals a novel family of telomere-associated lncRNAs
Source: Genome Biol. 2011 Jun 20;12(6):R56. doi: 10.1186/gb-2011-12-6-r56 (PMC3218844; doi:10.1186/gb-2011-12-6-r56)
Supplement: Additional file 16 — Probe hybridization intensity versus G+C content. Boxplots of intergenic probe hybridization intensities for each sample. Probes are grouped according to number of G+C bases. [file gb-2011-12-6-r56-S16.PDF]

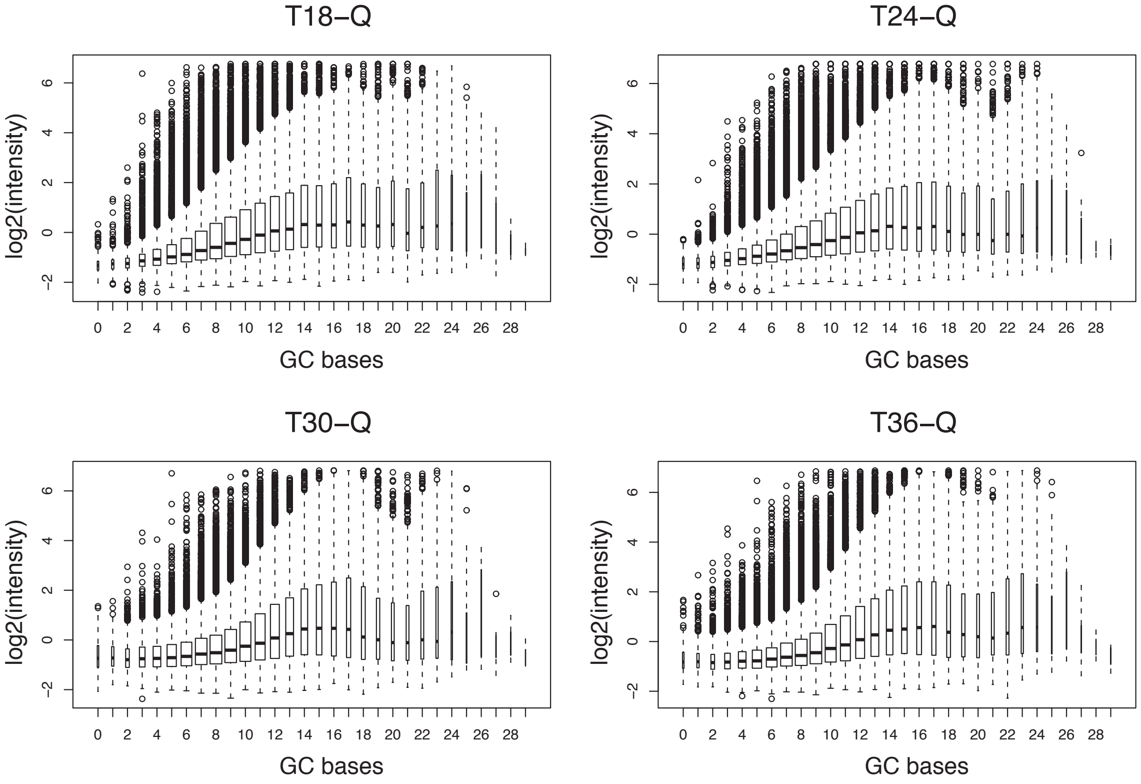

Figure A8. Minimal increase in median intergenic probe hybridiization intensity is observed with number of G+C bases. Boxplots of intergenic probe hybridization intensities for each sample after quantile normalization. Probes are grouped according to the number of G+C bases in their sequence.
